# An Eye-Tracking Study on Six Early Social-Emotional Abilities in Children Aged 1 to 3 Years

**DOI:** 10.3390/children11081031

**Published:** 2024-08-22

**Authors:** Thalia Cavadini, Elliot Riviere, Edouard Gentaz

**Affiliations:** 1 Department of Psychology, University of Geneva, 1205 Geneva, Switzerland; thalia.cavadini@unige.ch (T.C.); elliot.riviere@unige.ch (E.R.); 2 Univ. Lille, ULR 4072–PSITEC–Psychologie: Interactions Temps Emotions Cognition, F-59000 Lille, France; 3 Swiss Center for Affective Sciences, University of Geneva, 1205 Geneva, Switzerland; 4 Centre National de la Recherche Scientifique, F-38400 Grenoble, France

**Keywords:** social-emotional abilities, children, eye-tracking, visual preferences, social-emotional development norms, typical development

## Abstract

Background: The experimental evaluation of young children’s socio-emotional abilities is limited by the lack of existing specific measures to assess this population and by the relative difficulty for researchers to adapt measures designed for the general population. Methods: This study examined six early social-emotional abilities in 86 typically developing children aged 1 to 3 years using an eye-tracking-based experimental paradigm that combined visual preference tasks adapted from pre-existing infant studies. Objectives: The aim of this study is to obtain developmental norms in six early social-emotional abilities in typical children aged 1 to 3 years that would be promising for an understanding of disorders of mental development. These developmental standards are essential to enable comparative assessments with children with atypical development, such as children with Profound Intellectual and Multiple Disabilities (PIMD). Results: The participants had greater spontaneous visual preferences for biological (vs. non-biological) motion, socially salient (vs. non-social) stimuli, the eye (vs. mouth) area of emotional expressions, angry (vs. happy) faces, and objects of joint attention (vs. non-looked-at ones). Interestingly, although the prosocial (vs. antisocial) scene of the socio-moral task was preferred, both the helper and hinderer characters were equally gazed at. Finally, correlational analyses revealed that performance was neither related to participants’ age nor to each other (dismissing the hypothesis of a common underpinning process). Conclusion: Our revised experimental paradigm is possible in infants aged 1 to 3 years and thus provides additional scientific proof on the direct assessment of these six socio-emotional abilities in this population.

## 1. Introduction

The child’s development promotes the progressive acquisition over the first few years of life of a multitude of cognitive, emotional, social, and physical abilities [1]. This development is particularly important in the first three years of life, when growth and development are rapid, and is greatly shaped by the early social-emotional interactions that the young child has with his environment [2,3]. This development involves the acquisition of social and emotional abilities that progressively enable children to interact with and adapt to their social environment, to learn from it, to regulate and communicate their emotions, and to develop socially appropriate interactions and relationships with others [1,4]. These early social-emotional abilities thus appear to be essential precursors to health, well-being, and quality of life, as well as increasing cognitive ability, success at school, and community involvement [5].

The necessity of evaluating these abilities among toddlers is therefore indisputable [6]. Nevertheless, the manner in which this assessment is carried out with this population involves a number of challenges and confronts researchers with a whole host of limitations that prevent them from obtaining a true measure of these social-emotional abilities [6]. There is a lack of studies in the literature concerning the experimental evaluation of young children’s social-emotional abilities, which tends to be explained by the absence of existing specific measures for evaluating this population, which has no language and whose behavior is unpredictable and variable, as well as by the relative difficulty of researchers in adapting measures designed for the general population [7]. In light of these difficulties, the pursuit of studies seems to depend largely on researchers’ ability to develop innovative methodologies that would obtain data derived directly from the social-emotional behavior of young children.

The development of communication is underpinned by various early social-emotional abilities, which, when altered, have an impact on learning. These early abilities are of major importance in social and communicative development, and among them we might mention preferential attention to biological movement, social orientation, visual exploration of human faces and discrimination of facial expressions of joy and anger, joint attention, and socio-moral evaluations. Together, they support non-verbal communication and provide appropriate social stimulation, which is essential for the development of communicative intent [8].

The first ability assessed in the current study was *Preferential attention to biological motion*, which is one of the earliest perceptual abilities involved in the development of non-verbal communication (cf. [9] for a review). *Biological motion* refers to the movements produced by living vertebrate organisms that are characterized by dynamic regularities reflecting the structure and control patterns of the musculoskeletal system [10]. To investigate the ability to discriminate between biological and non-biological motion, Johansson [11] developed his “Point-Light paradigm” by strategically placing light markers on the major joints of an individual walking on a treadmill in the dark. He was thus able to model the movement of this walker as a sequence of light points moving according to human walking locomotion, which he could then present next to a second set of randomly moving points in order to test the preference for biological (vs. non-biological) motion. The human visual system is extremely responsive to others’ movements and is able to process and discriminate biological motion very quickly (100 milliseconds) [12]. Using similar paradigms, a large body of work has confirmed and demonstrated the existence of a spontaneous preference for biological over non-biological motion in humans but also in a wide range of animal species [13]. Moreover, this sensitivity appears very early in development: from the first days of life, human neonates preferentially direct their attention to this type of movement [10,14,15]. However, although this ability has already been studied in adults, children, infants of a few months of age, and newborns [16], little is known about its development between the first and third years of life.

As a continuation of the previous ability, we then decided to focus on *Social Orienting*, referring to the intrinsic ability to preferentially orient to our surrounding social environment [17]. Like preferential attention to biological motion, social orienting appears very early in development: from birth, newborns show a particular sensitivity to socially salient stimuli [18,19]. Moreover, this predisposition to orient preferentially to the social environment is of crucial importance early in social-communicative development [20]. Indeed, by orienting to peers, infants prompt further interaction, creating rich learning opportunities through reciprocal social engagement. This is supported by research in the field of autism that consistently reports impaired social orienting in children with ASD, a developmental disorder characterized by social and communicative impairments (cf. [21] for a review). Nevertheless, this ability seems to have been little studied in TD infants between the ages of 1 and 3 years.

Considering the topics already discussed, it seemed essential to us to focus next on the most salient social stimulus for humans: faces, and more precisely on the *Facial Expression Exploration* ability. From birth, newborns orient preferentially to faces rather than to any other object; as early as 9 min after birth, the newborns are already sensitive to the schematic (‘humanoid’) configuration of faces [22]. A few hours later, they are able to discriminate between very familiar faces, such as their mother’s, and unknown ones, even if they look alike [23]. From 12 h onwards, they can recognize unfamiliar faces (to which they had only been briefly exposed) [24]. Moreover, gaze plays a very important role in newborns’ early interest in faces; they look more at faces with open rather than closed eyes and also prefer to look at a face with eyes directed towards them rather than away [25]. Advances in eye-tracking technology have made it possible to study more precisely the visual exploration of internal (e.g., eyes, nose, mouth) and external (hairstyle and jaw) facial features and to demonstrate that both adults and children look at the eye area more than any other facial feature [26]. This preference has also been found in infants as young as three months of age [27], but again, there is little work after the first year of life until childhood. This specific exploration of faces plays a fundamental role in the development of social communication (cf. [28] for a review). In addition to the identity of individuals, faces provide us with relatively stable information about our social partners, allowing us to categorize a person, even an unknown one, according to different dimensions useful in social interactions (such as gender, age, or ethnicity), but also to be able to infer the affective and attentional states of others as well as their intentions, thanks to the analysis of facial features (cf. [29] for a review).

The fourth ability we examined was *Emotional Faces Discrimination*, focusing on the discrimination of anger and joy. A very large body of work has studied the typical development of discrimination in emotional facial expressions during the early years. There is a sensitivity to changes in facial expression as well as an attraction to happy faces from the first days of life (cf. [30] for a review). However, the latter is not systematically reported at that age because newborns’ discrimination abilities are limited by their low visual acuity and experience and are modulated by several environmental factors (e.g., familiarity, contrast, intensity, etc.) (cf. [31] for a review). This preference for smiling faces, observed under specific conditions from birth, persists and is perfected during the first months of life. Between 3 and 6 months of age, babies are progressively able to differentiate joy from other emotional expressions: at 3 months, they discriminate the expression of joy from anger (frowning) and surprise; they then succeed in differentiating joy from sadness between 3 and 5 months, and from fear at 4 months, whereas they are able to discriminate between joy and neutral expressions as well as between joy and surprise from 5 months [30]. Finally, it is only at 6–7 months that babies are able to distinguish between expressions other than smiling [32]. In contrast, the discrimination of emotional expressions after the first year has been rarely studied in the past.

We then focused on the ability to follow the gaze of others by studying *Joint Attention*, defined as a co-created outcome of an interaction in which two individuals’ shared interest in the same object (cf. [33] for a review). This is one of the earliest mechanisms by which a child can interact and learn (cf. [34] for a review). Essential to the development of non-verbal communication, this ability emerges very early: a dyadic relationship between parent and baby begins to be established from birth through mutual gaze exchanges and will gradually increase in both quantity (number of occurrences) and quality (integration of other communication modalities such as smiles, gestures, or vocalizations) during the following first months (cf. [35] for a review). Then, between 4 and 9 months of age, infants start to initiate spontaneous joint attention behaviors aimed at directing the attention of others toward a specific target [36]. Subsequently, they diversify the use of their own visual coordination to exhibit joint attention response behaviors that are aimed at attentional sharing, such as following the parent’s gaze to share a common source of interest. Thus, joint attention requires both expressive (i.e., *initiation* of joint attention behaviors) and receptive (i.e., *response* to joint attention behaviors; abbreviated here as RJA) abilities [37]. In the present study, we focused on RJA behaviors defined by Mundy et al. [36] as the ability to follow the direction of others’ gaze and gestures to share a common point of reference. Autism research has shown that RJA behaviors are generally impaired in ASD children, highlighting the great importance of this ability in social-communicative development [38]. Indeed, through triadic exchanges that promote involvement in social interactions, joint attention is the precursor of pointing, intention to communicate, and language [35].

The final *Socio-Moral ability* investigated was the capacity to evaluate others based on their prosocial or antisocial actions toward others [39]. Although moral judgment (studied with verbal tasks) follows a staged development that extends from infancy to adolescence [40], its foundations may be initiated very early. Indeed, it appears that infants are able to intuitively evaluate some social interactions long before the emergence of self-awareness, language, and theory of mind [41]. By creating the “Hill paradigm” in which a puppet trying to climb a hill is either helped in its action by another character (i.e., the ‘helper’ with a prosocial behavior) or prevented from reaching the top by another one pushing it down (i.e., the ‘hinderer’ with an antisocial behavior), Hamlin, Wynn, and Bloom [39] showed for the first time that infants were more attracted to prosocial actions (exhibiting reaching, grasping behaviors) as early as 6 months of age. By adding a visual preference procedure to this paradigm, they were even able to find that 10-month-old infants preferred to look at prosocial agents [39]. A preference that the authors later measured from the age of 3 months with this same paradigm [42] but also with a different one (the “Ball paradigm”): Hamlin and Wynn [43] observed that 3-month-olds preferred a puppet that gave its ball to another character (i.e., prosocial action) compared to one that took someone’s ball (i.e., antisocial action). The early manifestation of these socio-moral evaluations highlights that infants are able to interpret the moral valence of these scenarios and express a preference toward prosocial behaviors. Since the publication of the original study by Hamlin et al. [39], numerous replications have been conducted using various paradigm adaptations (cf. [44] for a review). However, the results of these studies are generally inconsistent and struggle to replicate the original findings (cf. [44,45] for reviews). In addition, there is little work on the age range of 12 to 36 months (i.e., the age range of interest in the present study). Using an adaptation of the “Ball paradigm”, Scola et al. [46] observed, for example, that both 12- to 24- and 24- to 36-month-olds showed a preference for prosocial actions, whereas Cowell and Decety [47] found no preference neither for prosocial nor antisocial actions with the “Hill paradigm” in 54 infants aged 12–24 months. 

### Objectives et Hypotheses

The aim of this study is to obtain developmental norms on six early social-emotional abilities from a large sample of typically developing (TD) children aged 12 to 36 months. To do this, it aims to use a revised version of an experimental paradigm based on eye-tracking that includes various visual preference tasks.

Since we used a revised version of an experimental paradigm designed initially to study the six abilities described above in both children with multiple disabilities and a small control group of TD children [8], the first objective of the current study was to ensure that the revisions made to the experimental paradigm had not affected its structure. We hypothesized that the percentages of looking time (LT) on the six blocks of visual stimuli presented would be independent of the order of presentation (Hypothesis 1a) and would not be subject to fatigue effects (Hypothesis 1b). Therefore, the average percentages of looking time for the first two blocks should be statistically equivalent to those of the last block. Additionally, we hypothesized that the content of the blocks would not influence the percentages of looking time (Hypothesis 1c).

The second objective of this study was to explore the relationships between the six social-emotional abilities studied. First, we hypothesized that the percentages of looking time for the tasks would be correlated with the overall percentage of looking time for the entire session (Hypothesis 2a) and that they would also be inter-correlated (Hypothesis 2b).

We then formulated several hypotheses regarding the correlations between the percentages of looking time and the children’s age. We expected age to be correlated with the percentages of looking time for the entire session (Hypothesis 3a) and for each individual task (Hypothesis 3b). We also hypothesized that the children’s age would be correlated with the difference in looking time between the two paired stimuli in each task (Hypothesis 3c), indicating their degree of discrimination. Furthermore, assuming that a common underlying process might drive the abilities studied, we hypothesized that the difference scores in looking time would be correlated with each other (Hypothesis 4).

Finally, we also expected to observe visual preferences, reflected by higher looking times, for biological motion compared to non-biological motion (Hypothesis 5a), socially salient scenes compared to non-social scenes (Hypothesis 5b), the eye region compared to the mouth region in facial expressions (Hypothesis 5c), angry faces compared to happy faces (Hypothesis 5d), objects of joint attention (Hypothesis 5e), the helping character compared to the hindering character (Hypothesis 5f), and the prosocial climbing scene compared to the antisocial scene (Hypothesis 5g).

## 2. Materials and Methods

In this study, we examined six social-emotional abilities that play a key role in later communicative development, namely: Preferential attention to biological motion, Social orientating, Facial expression exploration, Emotional faces discrimination (anger vs. joy), Joint attention, and Socio-moral evaluation. These were investigated in the same 86 typically developing (TD) 2-year-old children (aged from 12 to 36 months) using a revised version of the eye-tracking-based experimental paradigm we originally designed to improve the assessment of the psychological development of children with Profound Intellectual and Multiple Disabilities (PIMDs), which still remains a very challenging process in this clinical population [8,48]. Indeed, both the severity of the intellectual disabilities and the extent of the motor impairments that characterize this particularly heterogeneous population hinder the conduct of psychological assessments with these individuals. This clinical population is described as “a heterogeneous group of individuals characterized by very severe cognitive, neuromotor, and/or sensory disabilities, resulting in very intensive support needs” [4]. These individuals have a statistically estimated IQ below 20–25 and an overall developmental age of 24 months or less [49,50]. This experimental paradigm was therefore aimed at responding to the real need to develop new evaluation tools and methods to better identify their needs [4]. This paradigm included a panel of visual preference tasks inspired by preexisting infant studies, coupled with an eye-tracking device. This equipment was used to record participants’ spontaneous eye movements in response to the successive presentation of various pairs of visual stimuli on a screen.

Furthermore, our initial study not only examined the competencies of PIMD individuals; it also explored the social-emotional abilities of young TD children, as a limited control group of 32 2-year-olds (aged 12–36 months) had also been recruited in that first study to better understand the inter-individual variability observed in PIMD individuals by conducting case-control comparisons [8]. The absence of studies on this particular toddler population constitutes a significant gap in modern research, as it does not allow for the collection of information or the creation of control groups for comparison with atypically developing populations. Interesting previous analyses conducted with data from this limited control group reproduced the visual preferences observed in infant studies but also revealed some unexpected results. However, these were not investigated further because the earlier study focused on PIMD individuals and not on TD participants.

Additionally, this early study had highlighted some methodological limitations, so it was necessary to design a novel version of the paradigm that took them into account in order to confirm or not confirm the (unexpected) results obtained previously with a sample of only 32 TD 2-year-olds. For all these reasons, and in order to obtain robust developmental reference standards that would allow for comparative analyses with children with PIMD, we conducted the current study.

### 2.1. Participants

A total of 86 TD infants (38 girls and 48 boys) aged 12 to 36 months (mean age = 23 months, *SD* = 7.2 months) participated in the study. Study participants were required to be typically developing young children, aged 12 to 36 months, without neurodevelopmental disorders identified at birth and born at term. Every age between 12 and 36 months was fairly represented. Eight additional infants took part in this study but were excluded from the analyses because of procedural issues in data collection (mainly due to device disconnection, parental interventions, or infant distress/crying during the experiment). All participants were recruited through personal and professional networks between September 2021 and August 2022. Parents were contacted by means of an information letter in which all the characteristics of the study were presented, and they gave their informed written consent for their child to take part. The eye-tracking sessions carried out at the University of Geneva’s BabyLab were anonymized; no information identifying individual participants was collected. The study was conducted in accordance with the latest Declaration of Helsinki, and its experimental protocol had been approved by the Ethics Committee of the University of Geneva.

### 2.2. Apparatus and Visual Preference Procedure

Based on the principle that a gaze fixation on a specific point provides a measure of visual attention to various stimuli [51], eye-tracking technology has been widely used. In the present study, we used the Tobii Pro X3-120 binocular corneal-reflection (CR) eye-tracker (Tobii Technology AB, Stockholm, Sweden), which records at a gaze sampling frequency of 120 Hz. This device detects the position of the eyes and records the movements they make based on Pupil Center Corneal Reflection (PCCR). The PCCR eye-tracking technique uses the relative positions of the pupil and a reflection of a light source on the cornea (near-infrared light) to compute pupil positions (gaze). These reflections—the vector between the cornea and the pupil—are tracked by an infrared camera and fed into the computer software, where the pupil/CR relation is calculated with image processing algorithms, allowing a gaze estimate with a theoretical spatial resolution of about 1 visual degree [52].

This technology is particularly relevant in the field of infancy research, where such devices are increasingly used as measuring tools, especially because they provide great spatial and temporal precision [53]. By defining *Areas of interest* (AOIs), eye-tracking devices allow to examine, in a non-invasive way, where, when, in which order, and during which duration these areas have been specifically gazed, thus offering a detailed examination of individual visual exploration [54].

In addition, this device can be used as part of various experimental methods, such as habituation and response to novelty paradigms [55] or in the framework of a visual preference procedure [56], as was the case in the present study. Indeed, our experimental paradigm was composed of tasks using a classic fixed-trial visual preference procedure. This experimental method was first developed by Fantz [57] to study the perceptual abilities of infants. Combined here with an eye-tracking device, it consists of simultaneously presenting a person with two visual stimuli that differ from each other on one property (stimuli ‘A’ and ‘B’) placed side by side on either side of a presentation screen equipped with the eye-tracker and then comparing the time spent looking at the stimulus located on the left side of the screen versus the one on the right side. Usually, the visual scene formed by the pair of A–B stimuli is presented repeatedly (i.e., trials) by alternating their respective positions on the screen across the different trials in order to laterally counterbalance the stimuli’s locations and thus limit side bias. When a person spends, on average, more time looking at one of the two stimuli, it can be said that he or she has perceived a difference between the two stimuli presented. However, if they are equally gazed at, it is not possible to draw any conclusions; in this case, we cannot know whether the person perceived that they differed from each other or not.

### 2.3. Experimental Procedure

Each participant realized a single individual eye-tracking session at the University of Geneva’s BabyLab. Initially, the parents read and signed the consent form and filled out a brief sociodemographic questionnaire. The child was then seated comfortably in front of the screen by the accompanying parent, either in a suitable seat (*n* = 72) or on the parent’s lap. In the latter case, the parent placed opaque glasses on his face to avoid any interference with the recording of the child’s eye movements (*n* = 14). The presentation screen was a 22-inch screen measuring 47.5 × 30 cm and having a resolution of 1920 × 1080 px. The screen was placed at a distance of 60–65 cm from the child’s eyes and respected a visual angle of α ≃ 31.5° so as to comply with the recommendations of the eye-tracker user manual. All parents were instructed to remain motionless and not to intervene during the experiment (unless absolutely necessary). The eye-tracking session, whose content is detailed in the Stimuli section, could then begin. To minimize visual distraction, the light was kept at a low level throughout the experiment [58]. When the experiment was finished, the children received a medal as a thank you for participating.

A session included the following elements: (a) a 30-s opening cartoon (to focus participants’ attention), (b) a 5-point calibration procedure, (c) the experimental phase, and (d) a brief closing animation featuring a puppet thanking the participant and saying goodbye. The eye-tracker calibration procedure consisted of the appearance of an expanding and contracting white circle on a black background at each position of a grid and extracting five points from the four corners of the screen. The procedure was completed when all five points had been successfully calibrated and repeated for the missing points. Calibration was then considered satisfactory when it was obtained with a deviation of less than 2° on the X and Y axes. Various visual stimuli were presented successively in random order during the experimental phase (details are given in Stimuli section). The total duration of a session, including the successive sequence of the 4 steps presented above (the opening cartoon, the calibration, the experimental phase, and the final animation), was approximately 7–8 min.

### 2.4. Stimuli

To measure the social-emotional abilities of interest, we integrated six ‘blocks’ of experimental content into our paradigm (each underpinning one of these competencies). To implement a visual preference procedure involving the simultaneous presentation of two side-by-side stimuli, each task included at least two trials to laterally counterbalance the stimulus pairs. Each block and all trials were separated by the presentation of a dynamic fixation cross-paired with a stimulating sound in the center of the screen; its duration was fixed at 1.5 s between trials, whereas between blocks it had to be gazed at for at least 300 milliseconds (ms) for the next one to be triggered. This choice was based on the fact that shorter-duration fixations than the 300 ms threshold are commonly considered involuntary [59,60]. The activation area drawn around it was 400 × 400 px. The order of presentation of the six blocks, as well as that of their respective trials, was random.

Since we used a basic 5-point calibration procedure and, in addition, participants’ gaze was repeatedly redirected to the center of the screen, we decided not to record data on the area at the intersection of the two stimuli over the entire height of the screen. Therefore, when the two paired stimuli extended across the entire screen surface (as in PLM-, SO-, and SME-Tasks described in the next section), their respective positions were defined by two areas of 850 × 1080 px that were activated only after 300 ms of visual scene presentation. There was thus a central 220 × 1080-px strip in the middle of the screen, which was not covered by any AOIs and whose width was defined on the basis of the distance separating the pairs of emotional faces.

A. The Point-Light Motion (PLM) Task

Based on Johansson’s [11] “Point-light paradigm”, we designed our ‘PLM-Task’ to assess preferential attention to biological motion using the stimuli created by Bidet-Ildei et al. [15]. Biological motion was represented by dynamic points of light depicting the movement of a human walker (nine points: the head, one shoulder, one elbow, one wrist, one hip, both knees, and both ankles) over a second set of nine randomly moving points (non-biological motion). The amount of movement per point was equivalent between conditions [15]. This task consisted of four 10-s trials in which the lateral positions of the two stimuli on the screen were alternated. They were delineated by two 850 × 1080-px AOIs. Among the four trials, in two of them, the dots were moving from the center of the screen to the sides and from the sides of the screen to the center in the other two trials, alternating the lateral positions of the two motion types between each trial. Thus, all four possible conditions were presented to each participant, which was not the case in the previous study, where this task was composed of two 20-s trials (each direction was presented only once).

B. The Social Orienting (SO) Task

We designed the ‘SO-Task’ using stimuli created by Franchini et al. [61] based on the previous work of Pierce et al. [62] to evaluate the participants’ ability to discriminate socially salient visual scenes from non-social ones. This task comprised eight trials, each lasting 5 s, resulting in a total duration of approximately 48 s including the inter-trial intervals. During the trials, participants were presented with pairs of side-by-side stimuli: dynamic social images (sequences showing a young boy or girl moving and dancing alone) and dynamic geometric images (moving geometric shapes resembling traditional abstract screen savers). They were delineated by two 850 × 1080-px AOIs. The structure of the paired-comparison stimulus display was controlled by the authors who initially designed this task: the stimuli were equal in visual salience independently of social/non-social conditions; the stimulus brightness was also equivalent between conditions; as was the area of moving elements [61]. Compared to the previous study [8], no changes were made to this task, which remained identical here.

C. Emotional (angry and happy) faces

Since two competencies were studied with only one type of stimulus (Facial expression exploration and Emotional faces discrimination), we decided to dedicate two blocks to these stimuli, one consisting of a pair of emotional expressions (anger and joy) of a female model and the other of a male model, in order to have the same number of blocks as competencies and thus avoid any confusion. The faces used were taken from the Karolinska Directed Emotional Faces database (KDEF) [63], references F22 and M17. This selection was informed by the validation study of this database [64], which identified the only models among the top 20 joy and anger pictures with visible teeth. The presence of teeth is a salient facial feature that significantly influences emotion discrimination [65]. To enhance ecological validity, the stimuli were displayed in color against a medium gray background (RGB 100, 100, 100) [66]. Additionally, the hairline, a facial feature known to impact visual face exploration [24], was cropped using GIMP software (Version 2.10).

Each of the two experimental blocks devoted to emotional faces consisted of four 8-s trials. Since the order in which they were presented was random, it was possible for these blocks to be presented successively in some of the many individual sessions we conducted with our participants. during some of the numerous experimental sessions. However, as they consisted of static and silent stimuli, they were likely to be less attractive than the other dynamic stimuli we used and were therefore likely to decrease the participants’ visual attention. To prevent this, the presentation duration for each trial is slightly shorter than for the other tasks.

Different AOIs were defined as the two abilities were actually measured by comparing the time spent looking at different locations: (a) for visual scanning of facial features, the time spent on the *eye area* (composed of two AOIs of 215 × 215 px each, spaced 80 px apart) was compared to the time spent on the *mouth area* (consisting of an AOI of 430 × 215 px, equivalent to the size of the eye area), and (b) for emotion discrimination, the time spent on *angry faces* was compared to that spent on *happy faces* (defined by two 740 × 980-px AOIs, symmetrically positioned at a distance of 110 px from the screen center). The size of the faces (and consequently those of the different AOIs as well) was larger than in the previous study in order to maximize the data recorded on each of them, which tended to be very low (especially in PIMD individuals).

D. The Response to Joint Attention (RJA) Task

The ‘RJA-Task’ of our paradigm used an adaptation of the stimuli created by Franchini et al. [38] to test participants’ ability to follow the direction of others’ gaze to share a common point of reference. We included in our paradigm only the videos of the ‘intense’ condition, which had previously produced significantly longer viewing times. These comprised four videos lasting 12 s each in which an actress stood behind a table with two identical objects (differing in nature, shape, and color in each video). In this initial position, the actress gazed directly at the camera. After 2 s, the objects started to move, and she unexpectedly directed her attention to one of them with an expression of intense surprise for the remaining 10 s. The objects’ location was defined by two 860 × 500-px AOIs (symmetrically located at a distance of 50 px from the center of the screen length), whereas the actress’ head was delimited by an additional AOI of the same size. This third area was of great importance because it had to be gazed at for at least 500 milliseconds (i.e., the minimum fixation duration threshold for visual processing of dynamic scene information in free-viewing conditions, according to Glöckner and Herbold [67]) for a trial to be validated and included in the analyses. All AOIs were only activated when the actress started to be surprised by one of the objects (i.e., 2 s after the video started) for a duration of 10 s.

In the original stimuli (i.e., such as those used in the first version of our paradigm), there was a whole empty space around the table and the actress that we cropped for the present study. Thanks to this ‘zoom’, the actress’ face was then much larger, and thus her expression of surprise was more easily detectable. Consequently, the AOIs were larger than before and should thus contribute to a lower loss of data (i.e., including more valid trials in the analyses).

E. The Socio-Moral Evaluation (SME) Task

The ‘SME-Task’ of our paradigm was adapted from the “Hill paradigm” [39]. In this task, a ‘climber’ puppet (represented by a red and circular wooden character with large plastic ‘googly’ eyes) repeatedly attempted to climb a steep hill but failed. The climber was then either helped up the hill by a ‘helper’ puppet who pushed him upwards (demonstrating prosocial behavior) or pushed down by a ‘hinderer’ puppet (demonstrating antisocial behavior). These helper and hinderer roles were counterbalanced over two contrasting stimulus shapes: a blue square and a yellow triangle, both with googly eyes. Each of these ‘climbing scenes’ lasted 15 s. Following these scenes, a final visual preference scene was presented, consisting of two 15-s trials in which both the helper and the hinderer puppets were simultaneously displayed side by side, occupying the entire screen surface, which was delineated by two 850 × 1080-px AOIs. Thus, unlike the other tasks in our study, the SME-Task included a visual preference scene only after the initial presentation of the first two climbing scenes. In addition, participants’ socio-moral evaluation ability was also measured by comparing the time spent gazing at each climbing scene to determine whether one of the two observed behaviors (pro- vs. anti-social) attracted more visual attention.

### 2.5. Data Analysis

Eye-tracking data was extracted using Tobii Pro Lab (Version 1.162) computer software, which obtained metrics on ‘*total visit duration*’ (i.e., total time each AOI was gazed) within all the designated AOIs as well as within the screen area. From the visit durations within the AOIs, total dwell times (TDTs) were calculated by summing up all dwells (set of one or more consecutive fixations in an AOI) recorded during each task’s trials (excluding the invalidated ones of the RJA-Task), whereas visit durations within the screen area were converted into looking time (LT) percentages by computing the ratio between the time spent looking at the screen regarding the blocks, tasks, and the entire session over their respective presentation durations. These percentages were used for investigating our first hypotheses that, independently of their content, the six blocks presented would be equally gazed at. Indeed, we expected that the six blocks’ LT percentages (i.e., time spent looking at the screen during the presentation of each block divided by the block duration) would neither be influenced by (1a) the order of presentation nor by (1b) a fatigue effect (i.e., the average LT percentages of the first two blocks presented would be statistically equivalent to those of the last). Similarly, we hypothesized that each type of visual stimulus would attract children’s attention in equal measure and consequently that their LT percentages on the five experimental tasks (calculated as the proportion of time spent looking at the screen during the presentation of each task over the task duration) would not be influenced by the (1c) content effect.

Two one-way repeated measures ANOVAs were performed to test these hypotheses about the paradigm conception. The first one investigated the effect of presentation order by entering blocks’ LT percentages as a 6-modality within-subjects factor, while the fatigue effect was examined through a post-hoc contrast analysis (average LT percentage of blocks 1 and 2 vs. of blocks 5 and 6). The second ANOVA was then conducted with tasks’ LT percentages entering as a 5-modality within-subjects factor (given that LT percentages within both female and male emotional faces were averaged and considered as a single type of content) for testing the content effect. Tukey HSD tests were run post-hoc when the results of these analyses were significant (*p*-value ≤ 0.05).

LT percentages were also used to examine the links between the abilities assessed through correlational analyses. Thus, Pearson correlations were conducted to investigate our second hypotheses that tasks’ LT percentages would (2a) correlate with the overall session LT percentage (calculated as the proportion of time spent looking at the presentation screen over the session duration) since visual attentional focus increases with age [68] and (2b) intercorrelate with each other. Thirdly, we also made several hypotheses about the links between these LT percentages and children’s age: we expected that age would correlate (3a) with the overall session LT percentages as well as (3b) with each task’s LT percentage. In addition, we expected that children’s age would correlate (3c) with the difference between the time spent looking at each of the two paired stimuli in each task (gaze time difference scores were thus calculated by subtracting the TDTs within both paired stimulus types of each task, which quantifies how well discriminated they were). Moreover, assuming that a common process might underlie the competences studied, we hypothesized that (4) these differences would correlate with each other.

Finally, regarding visual preferences, based on our previous findings, we expected to observe preferences resulting in higher TDTs on (5a) biological (vs. non-biological) motion, (5b) socially salient (vs. non-social) scenes, (5c) the facial area of the eyes (vs. mouth), (5d) angry (vs. happy) faces, (5e) objects of joint attention, and (5f) the helper (vs. hinderer) character, as well as (5g) the prosocial (vs. antisocial) climbing scene. These hypotheses were tested by conducting paired-difference *t*-tests to compare whether TDTs within the two paired stimuli of each task were statistically equivalent, in which case no conclusion could be drawn (since it was impossible to determine whether their distinction had been perceived or not). However, when the analysis revealed that they differed significantly from each other, it was possible to conclude that there was a visual preference for one of them (i.e., for the stimulus with the highest TDTs) and thus to consider that they were discriminated against. Only the SME-Task (more precisely hypothesis 5g) required a special analysis: while the other tasks presented both types of paired stimuli simultaneously, the first two climbing scenes of the “Hill paradigm” were presented successively. Therefore, in addition to the paired *t*-test conducted with data from both trials of the final visual preference scene of this task, an independent *t*-test was also conducted to compare the visit durations within the first two climbing scenes.

## 3. Results

### 3.1. Participant Characteristics

An a priori sample analysis was conducted for paired-difference *t*-test statistical analysis with a moderate effect size of 0.4 and a *p* value of 0.05. Results (G-Power, Version 3.1.9.7) showed that the sample size suitable for this type of criteria was 84. All the infants were full-term and healthy. They were Caucasian and lived in rural areas (<3000 inhabitants; *n* = 16), villages (3000–10,000 inhabitants; *n* = 13), and urban areas (>10,000 inhabitants; *n* = 57) in the surroundings of Geneva, Switzerland. They came from lower- to upper-class families whose socioeconomic status (SES), expressed as a percentage using the formula of the Swiss author Genoud [69], ranged from 15 to 90% with an average of 68.22% (*SD* = 14.65). Parental age ranged from 22 to 55 years (*M* = 34.83, *SD* = 5.44).

### 3.2. Paradigm Structure Control Analyses

First, an absence of significant effects related to the presentation of the six blocks of experimental content on their respective LT percentages (order and fatigue effects) was found in the current study in line with what was already observed in the previous paper using a similar experimental paradigm: the repeated-measures ANOVA testing the main effect of the blocks’ presentation order was not significant [*F*(5,425) = 0.415, *p* = 0.838], as was the contrast analysis result comparing the mean LT percentages of blocks 1–2 vs. 5–6 to test the fatigue effect [*t*(85) = −0.846, *p* = 0.400].

The second within-subjects ANOVA revealed a significant effect of content on tasks’ LT percentages (calculated as the proportion of time spent looking at the screen during each task’s completion over their respective durations where both female and male emotional faces’ LT percentages were averaged and reported as the “emotional faces LT percentage” since they were considered to be a single stimulus type), *F*(4,340) = 27.466, *p* < 0.001. Multiple post-hoc comparisons (Tukey HSD test) revealed that both the SO-Task and the SME-Task, whose respective mean LT percentages of 84.104% (*SD* = 7.170) and 85.104% (*SD* = 8.725) were statistically equivalent to each other (*p* = 0.962), were significantly more gazed than the other three stimulus types (all *p*-values ≤ 0.001). Similarly, LT percentages for tasks assessing both preferential attention to biological motion (*M* = 76.143%, *SD* = 17.013) and response to joint attention (*M* = 78.334%, *SD* = 11.881) were also equivalent (*p* = 0.577), whereas the emotional faces were the least attractive type of experimental content, with the lowest mean LT percentage measured of 72.003% (*SD* = 11.193), which differed significantly from those of the other four task types (all *p*-values < 0.05).

### 3.3. Correlational Analyses

Regarding the correlational analyses, we were first interested in determining whether high LT percentages within each task-specific content type were associated with a high overall session LT percentage. Pearson correlations (detailed in Table 1) revealed that the five LT percentages measured within the different types of experimental content all correlated significantly with the overall session LT percentage (all correlations were significant at *p* < 0.01). The lowest correlation was for the task-specific stimuli of social orientating ability (*r* = 0.476) and the highest for those of response to joint attention (*r* = 0.803).

We then looked at the connections between the LT percentages within each of the five types of experimental content to investigate whether participants who looked a lot at the screen during the presentation of a certain type of content also looked a lot at the stimuli from the other tasks. All intercorrelations calculated were significant except one: the correlation between PLM- and SO-Tasks’ LT percentages (*r* = 0.135) was not significant (cf. Table 1). However, despite significant correlations, SO-Task’s LT percentage appeared to be less strongly related to that of both emotional faces and SME-Task (with respective correlations of 0.260 and 0.261, both significant at *p* < 0.05), whereas the intercorrelations between the LT percentages within the other tasks were all significant at *p* < 0.01.

The result of the analysis performed to test hypothesis 3a (correlation between participants’ age and overall session LT percentage) was not significant (*r* = 0.046), indicating that the amount of time spent looking at the screen throughout the experimental session was not associated with the age of the participants. The correlation matrix generated to test hypothesis 3b that older children would be more likely to have higher LT percentages within the different types of experimental content than the youngest showed that four of the five correlations computed were not significant (cf. Table 1). The only exception was the negative correlation between age and SO-Task’s LT percentage (*r* = −0.283, *p* < 0.01).

Finally, the results of the correlational analysis performed to test hypothesis 3c that age would be related to the ability to discriminate both types of paired stimuli in each task (TDTs difference score) did not reveal any significant correlation (cf. Table 2). Meanwhile, the correlation matrix resulting from this last analysis provided the intercorrelation values between the task-specific discrimination scores that hypothesis 4 assumed to be high (presuming the existence of a common process underlying the different competencies studied). It showed only one significant result, indicating that infants who looked more at the eye area (vs. mouth) of emotional faces also tended to look longer at socially salient stimuli (i.e., children dancing solo) rather than at non-social scenes (i.e., moving geometrical shapes) of the SO-Task (*r* = 0.343, *p* < 0.01).

### 3.4. Visual Preferences Analyses

With regard to visual preferences, we found the following significant results (cf. Table 3):

(a) Biological motion (mean TDT = 16.988 s, *SD* = 5.922) was preferred to non-biological motion (*M* = 14.617, *SD* = 4.957), *t*(85) = 2.641, *p* = 0.011.
(b) Socially salient stimuli (*M* = 27.012, *SD* = 4.921) were preferred to non-social ones (*M* = 9.826, *SD* = 4.851), *t*(85) = 16.712, *p* < 0.001.
(c) The eye area TDT (*M* = 9.678, *SD* = 5.287) was significantly higher than TDT within the mouth area (*M* = 5.701, *SD* = 4.799), *t*(85) = 4.970, *p* < 0.001.
(d) Angry faces (*M* = 15.070, *SD* = 4.256) were preferred to happy ones (*M* = 12.637, *SD* = 4.006), *t*(85) = 5.703, *p* < 0.001.
(e) Joint attention objects (i.e., those that were looked at by the actress) (*M* = 9.972, *SD* = 4.284) were preferred to non-looked-at ones (*M* = 5.715, *SD* = 3.002), *t*(85) = 10.141, *p* < 0.001.

**Table 3:** Total dwell times (*M*, *SD*) within the two stimuli types (A and B) of each task and statistical analysis (paired-difference *t*-tests) testing visual preferences (*N* = 86).

	Stimulus A		Stimulus B		Difference	
	*M*	*(SD)*	*M*	*(SD)*	*t*(*n* − 1)	*p*
Point-light motion (PLM-Task) ^1^	16.988	*(5.922)*	14.617	*(4.957)*	2.641	0.010
Social orienting (SO-Task) ^2^	27.012	*(4.921)*	9.826	*(4.851)*	16.712	<0.001
Facial Expression Exploration ^3^	9.678	*(5.287)*	5.701	*(4.799)*	4.970	<0.001
Emotional Faces Discrimination ^4^	15.070	*(4.256)*	12.637	*(4.006)*	5.703	<0.001
Joint attention (RJA-Task) ^5^	9.972	*(4.284)*	5.715	*(3.002)*	10.141	<0.001
Socio-moral evaluation (SME-Task) ^6^	9.731	*(3.463)*	9.610	*(3.190)*	0.265	0.791

^1^. Stimulus A = *Biological (walker) motion*; stimulus B = *Non-biological (scrambled) motion*. Total dwell times (TDTs) were computed by summing the four 10-s trials composing the PLM-Task. ^2^. Stimulus A = *Social scenes*; stimulus B = *Non-social scenes*. TDTs were computed by summing the eight 5-s trials composing the SO-Task. ^3^. Stimulus A = *Eyes area*; stimulus B = *Mouth area*. TDTs were computed by summing the total of eight 8-s trials of the evaluation of both Facial Expression Exploration and Emotional Faces Discrimination. ^4^. Stimulus A = *Angry faces*; stimulus B = *Happy faces*. TDTs were computed by summing the total of eight 8-s trials of the evaluation of both Facial Expression Exploration and Emotional Faces Discrimination. ^5^. Stimulus A = *Looked-at objects*; stimulus B = *Non-looked-at objects*. TDTs were computed by summing the four 10-s trials composing the RJA-Task. ^6^. Stimulus A = *Prosocial puppet (‘helper’)*; stimulus B = *Antisocial puppet (‘hinderer’)*. TDTs were computed by summing the two 15-s trials composing the final visual preference scene of SME-Task.

Regarding socio-moral evaluations, when both the helper and the hindered puppets were presented side by side simultaneously (final visual-preference scene), results showed no significant difference between the TDTs on the helper that had previously performed a prosocial action towards the climber by pushing him up the hill vs. on the hindered that had previously exhibited an anti-social action by pushing the climber down, *t*(85) = −0.605, *p* = 0.548. In contrast, when comparing the time spent looking at each of the first two successive climbing scenes, the analysis revealed that the prosocial scene was looked at significantly longer (mean TDT = 11.104 s, *SD* = 0.992) than the antisocial scene (*M* = 10.783, *SD* = 1.088), independent *t*-test: *t*(170) = 2.023, *p* = 0.045.

## 4. Discussion

The primary objective of this study was to extend scientific knowledge about six social-emotional abilities by testing them altogether in a large sample of 86 young TD 2-year-old (from the age of 12 to 36 months) children using a revised version of an eye-tracking-based experimental paradigm consisting of different visual preference tasks inspired by existing infant studies. As task-specific adjustments had been made to the original paradigm to address the methodological limitations previously highlighted, the present study first sought to ensure that its structure had not been affected by these revisions before focusing on the six competencies studied.

Regarding the analyses controlling the structure of the paradigm design itself, the fact that we found no effect related to the blocks’ presentation influencing LT percentages (neither order nor fatigue effects were found) demonstrated that the duration of the revised paradigm was appropriate for the population studied, thus supporting its feasibility. On the other hand, the significant effect of the content was novel and unexpected. It should first be noted that this unpredicted effect can hardly be attributed to the adjustments made to the original paradigm because they involved only certain task-specific stimuli and trials, i.e., all tasks had the same duration as initially. Moreover, the number of tasks was unchanged, as was the total duration of an experimental session, which was the same as in our previous study. Furthermore, although several potential explanations can be suggested for the low LT percentage of facial expressions, the high gaze LT percentages observed in both the SO- and SME-Tasks are difficult to interpret since no previous research has reported similar results. Indeed, the fact that facial expressions are a type of stimulus that is silent, static, and, moreover, has redundant content (the same pair of faces presented four consecutive times by simply alternating their lateral position between trials) has a negative impact on the attractiveness generated by this type of content (especially in comparison with dynamic and diversified stimuli such as those of the SO-Task, where the visual scenes of each of the eight trials are all different from one another). Not surprisingly, facial expressions were already the stimulus type for which the lowest LT percentage was measured in the previous study. Nevertheless, using only tasks composed of many short-duration trials, such as the SO-Task, would not be a suitable solution for the PIMD individuals we are still attempting to assess in a parallel case study who need much more time to process the information due to their extensive cognitive impairments. It is therefore better to use other strategies to increase the attractiveness of this type of stimulus. An example could be adding a third emotion and testing the ability to discriminate between joy/anger, sadness/anger, and joy/sadness. It would then be possible to present each of these three pairs of emotions only once in a first block and a second time in another block in order to alternate their lateral position on the screen while displaying short duration blocks. Different models could also be used in the two blocks to maximize the variability of the content presented in order to promote the attractiveness evoked by these static images, even though they are not dynamic scenes.

Next, with regard to visual preferences, we sought to extend the empirical knowledge and the developmental references on the six studied competencies between the ages of 1 and 3 years by implementing a revised version of our eye-tracking-based experimental paradigm with a new and larger sample of 2-year-old TD children. We were then interested in investigating whether the results of our previous study could be easily replicated and whether the modifications we made to the paradigm were beneficial.

First, all significant visual preferences that were observed in our previous study were also measured in the present research (i.e., preferences for socially salient stimuli, for the facial eye region, for angry faces, and for objects of joint attention).

Subsequently, some adjustments proved to be beneficial. In the present study, we found a preference for biological over non-biological motion (whereas these two motion types were statistically equal in the previous study). Given that visual processing of biological motion occurs extremely rapidly (100 ms) in humans [12], we presumed that our initial PLM-Task composed of only two 20-s trials was not suitable for assessing preferential attention to biological motion. Indeed, we hypothesized that the lack of significant difference between the TDTs within each of the two motion types could be related to an excessive duration of stimulus presentation, assuming that participants might preferentially direct their attention to biological motion during the first seconds of presentation but would then also tend to explore the moving dots of the non-biological motion, whose movements, close to those of a walker, violated the expectations depicting human walking locomotion. Therefore, we decided to present these stimuli for half the time by designing a revised PLM-Task consisting of four 10-s trials. It appeared that these modifications were useful since the present study revealed a significant preference for biological motion. This result extends the recent findings of the longitudinal study by Sifre and colleagues [70], which followed 116 infants over 2 years. With our sample of 2-year-old TD children with an age range up to 36 months, our results suggest that the linear increase in preferential attention for biological motion observed by Sifre et al. [70] between the ages of 2 and 24 months seems to continue throughout the third year of life. By providing evidence on the development of the ability to discriminate between biological and non-biological movement between the ages of 1 and 3 years, our findings contribute to filling the gap in the existing scientific literature on this age group. Indeed, although several studies have demonstrated the early emergence of this perceptual ability, showing that humans newborns already exhibit a preference for biological motion from birth on the one hand [10,14,15] and, on the other hand, developmental studies beyond infancy have shown that 5-year-olds [71,72] and even 4-year-olds [73,74] are as sensitive as adults to biological motion in point-light displays, few papers have provided any insight regarding the age range studied here (cf. [75] for a literature review on developmental trajectories).

The second modification that proved to be useful concerns the RJA-Task: although the benefits cannot be observed in terms of the results themselves (significant preference for joint attention objects found in this study as in the previous one), they can be observed in the quality of the recorded data. While we noted a substantial loss of data previously (many trials did not meet the inclusion criteria), no trials had to be excluded from the analyses in the present study. a large portion of the empty space surrounding the table and the actress, we were able to enlarge the four video sequences of the RJA-Task. The actress’ expression of surprise was then more salient. Moreover, all AOIs were larger, maximizing the opportunities to record gaze time on the one delimiting the actress’ face, which had to be gazed at least 500 ms for a trial to be validated.

Lastly, regarding socio-moral evaluations, our previous—somewhat conclusive—results appeared to be confirmed as the current study revealed similar findings: while the prosocial climbing scene attracted more visual attention than the antisocial one, no visual preference was measured when both the helper and the hinderer puppets were then presented side by side. Although we have not made any specific modifications to this paradigm since our first study, we were nevertheless particularly attentive to the experimental design of this task and carefully controlled that both the order in which the two scenes were presented and the pro- vs. anti-social role of each puppet were well randomized across participants. Unfortunately, by the end of our first study, we found that the randomization performed by Tobii Pro Lab software was approximate (the different conditions were not equally distributed within our previous sample of 32 individuals). Despite a more accurate randomized experimental design than previously, the analyses performed in the current study did not lead to more conclusive results. Such findings thus highlight the limited replicability of previous research using socio-moral scenarios similar to the “Hill paradigm” in young children and the difficulty, already discussed by several literature reviews, of replicating the results of the primary studies [44,45].

The secondary objective of the current study was to investigate the links between the six social-emotional competencies by conducting correlational analyses that revealed several interesting findings. First, we observed that, as expected, the tasks’ LT percentages correlated with the overall session LT percentage, indicating that the participants with the highest overall session LT percentages would be those who gazed the most at the screen during the different tasks’ completion. Similarly, the LT percentages of the different tasks tended to correlate with each other as well. Only the PLM- and SO-Tasks’ LT percentages appeared not to be associated. It is also interesting to note that, despite significant correlations, the analyses showed that the SO-Task’s LT percentage was weakly related to the LT percentages measured on the emotional faces as well as those of the SME-Task. Interestingly, these results can be linked to the content effect discussed above; indeed, the post-hoc multiple comparisons showed that emotional faces were the type of content on which the lowest LT percentages were measured and that, conversely, SO- and SME-Tasks were the ones on which the content attracted the most attention from participants. Thus, the results of the correlational analyses were consistent with those revealed by the analyses controlling for paradigm structure, highlighting the particular interest aroused specifically by these two types of content in our participants.

We were then interested in the role of participants’ age in these correlational analyses. The first surprising result was that there was a significant negative correlation between age and the LT percentage of the SO-Task, whereas the correlations between age and the other tasks’ LT percentages were all close to zero. This finding suggested that younger children tended to look more at the content of the SO-Task than older children. We then focused on the children’s performance on the different tasks assessed. Surprisingly, it appeared that the ability to discriminate between the different types of paired stimuli was not related to age. Indeed, the correlations between the participants’ age and their TDT difference scores on each task (quantifying discrimination abilities) were all close to zero. This indicated that the children who discriminated the different types of stimuli the best (i.e., those with the highest difference scores) were not necessarily the oldest. The correlation matrix generated also revealed that these discrimination abilities were not related to each other. Only preferring socially salient stimuli to non-social scenes in the SO-Task correlated significantly with looking more at the eye area of emotional faces than at the mouth. This association can be explained by the infants’ reliance on the eye area in order to obtain information for social interactions [25]. Initially, these intercorrelations were computed with the aim of investigating the potential existence of a common process underpinning the six abilities assessed here. The outcomes that had been highlighted by these analyses did not provide support for this hypothesis. However, assessing a set of six social-emotional abilities in the same sample of 86 children was unprecedented and therefore deserves to be studied in greater depth. Future work using this type of within-subjects experimental design in various populations would thus contribute to a better understanding of the associations between these abilities.

### Limitations and Implications

In our study, we obtained promising behavioral results by using an experimental paradigm based on eye-tracking combined with various visual social-emotional preference tasks, enabling us to further explore the social-emotional abilities of young children aged 2 years. We have shown that the proposed experimental paradigm is sufficiently sensitive to establish individual competency profiles, thereby making a contribution to developmental methods for assessing social-emotional abilities in young children.

The exclusive utilization of these computer-based behavioral tasks coupled with an eye-tracker, although effective in capturing children’s visual preferences and immediate and spontaneous reactions, has the main limitation of not necessarily offering a complete and contextual perspective of these social-emotional abilities. In fact, as we discussed earlier, other methods are used by researchers to assess these abilities (e.g., parent-reported questionnaires, observation methods, habituation/response to novelty techniques, etc.), which, in a complementary manner, could provide a more reliable and exhaustive measure of the social-emotional abilities of 1–3 year olds. In future studies, it could be interesting to combine the children’s visual behavioral preferences with hetero-questionnaires reported by the parents or professionals accompanying the children.

In this perspective, we used this complementarity of approaches with PIMD children by combining our experimental paradigm with questionnaires hetero-reported by professionals [8]. This combination of approaches revealed significant links between visual preferences (behavioral data) and hetero-reported responses (scores obtained on different subscales of the ECP [Évaluation-Cognition-Polyhandicap], a cognitive skills assessment scale for people with PIMD developed by Poujol et al. [76]), thereby strengthening the validity and consistency of these measures to provide a more holistic understanding of social-emotional abilities. The results of these two studies are very promising in the sense that they show that it is possible to use tasks initially developed to study the early abilities of babies to evaluate the social-emotional development of young children aged 1 to 3 as well as that of people with PIMD.

Our results with PIMD individuals thus suggest that the integration of varied methods may be essential to ensuring a comprehensive and nuanced assessment of young children’s social-emotional abilities. By diversifying methodological approaches, researchers can not only obtain more accurate data but also better understand the nuances and dynamics of social-emotional abilities in this critical age group. Consequently, future research should consider multi-method study designs to refine the assessment of social-emotional abilities in young children.

## 5. Conclusions

To conclude, in addition to providing further scientific evidence on six social-emotional abilities in the same 2-year-olds, the current study revealed that their respective performances were not related to each other either: only the preference for socially salient (vs. non-social) stimuli was significantly correlated with the preference for the eye area (vs. mouth) of emotional faces. Finally, this research investigated the replicability of a previous study that employed the original form of the eye-tracking-based experimental paradigm we used here. While all of the results that were significant in the first study were easily replicated in the current one, it also highlighted that the revisions made to the original paradigm contributed to more conclusive results and less data loss. Altogether, these findings demonstrated that the feasibility of the revised paradigm was greater than previously, making it a more suitable instrument for assessing clinical populations that are difficult to test, such as PIMD individuals. Indeed, direct assessment of these abilities in such atypical populations that have very specific and limited cognitive skills is, by nature, very difficult. At this moment, evaluation relies mainly on observational measures. Our results show that direct assessment of social-emotional abilities using our revised experimental paradigm is possible in infants aged 1–3 years and should therefore also be possible in PIMD individuals with an equivalent estimated developmental age. More recently, we also showed the relevance of this experimental paradigm to test the beneficial effects of innovative person-centered training promoting social-emotional abilities in these individuals [77]. This contributes to our scientific capacity to evaluate these abilities and our knowledge of the needs of this population.

## Figures and Tables

**Table 1:** Correlation matrix (Pearson’s *r*) between participants’ age (in months), overall session’s looking time (LT) percentage, and the five tasks’ LT percentages (*N* = 86).

	1	2	3	4	5	6
1. Age in months						
2. Overall session	0.046					
3. PLM-Task	0.094	0.718 **				
4. SO-Task	−0.283 **	0.476 **	0.135			
5. Emotional faces	0.185	0.649 **	0.356 **	0.261 *		
6. RJA-Task	−0.056	0.803 **	0.340 **	0.486 **	0.340 **	
7. SME-Task	0.082	0.710 **	0.367 **	0.260 *	0.321**	0.565 **

Note. * *p* < 0.05; ** *p* < 0.01.

**Table 2:** Correlation matrix (Pearson’s *r*) between participants’ age (in months) and total dwell time (TDT) difference scores on each of the two paired stimulus types in each task (*N* = 86).

	1	2	3	4	5	6	7
1. Age in months							
2. PLM-Task’ TDTs diff. score	0.109						
3. SO-Task’s TDTs diff. score	0.055	−0.005					
4. Face exploration TDTs diff. score	0.127	0.058	0.343 **				
5. Emotion discrimination TDTs diff. score	0.094	−0.066	0.149	0.168			
6. RJA-Task’s TDTs diff. score	0.002	−0.085	0.058	0.033	−0.012		
7. SME-Task’s TDTs diff. score (actions) ^1^	0.052	0.100	−0.053	−0.056	0.124	−0.029	
8. SME-Task’s TDTs diff. score (puppets) ^2^	−0.063	0.053	−0.096	−0.030	−0.019	0.070	−0.036

Note. ** *p* < 0.01. ^1^. Difference score between the TDTs on each of the two successive climbing scenes of the SME-Task. ^2^. Difference score between the TDTs on each of the puppets presented simultaneously in the final visual preference scene of the SME-Task.

## Data Availability

The dataset generated and analyzed during the current study is available in the Supplementary Data file.

## Supplementary Materials

The following supporting information can be downloaded at: https://www.mdpi.com/article/10.3390/children11081031/s1, Supplementary Data file. Cavadini, Riviere & Gentaz 2023 database.
